# Diet–Microbiome–Redox Interactions and Oxidative Stress Biomarkers in Livestock: Computational and Spatial Perspectives for Translational Health and Production

**DOI:** 10.3390/ijms27062556

**Published:** 2026-03-11

**Authors:** Paweł Kowalczyk, Apoloniusz Kurylczyk, Andrzej Węglarz, Joanna Makulska

**Affiliations:** 1 Department of Animal Nutrition, The Kielanowski Institute of Animal Physiology and Nutrition, Polish Academy of Sciences, Instytucka 3, 05-110 Jabłonna, Poland; 2 Institute of Spatial Management and Socio-Economic Geography, University of Szczecin, ul. Papieża Jana Pawła II 22a, 70-453 Szczecin, Poland; apoloniusz.kurylczyk@usz.edu.pl; 3 Department of Genetics, Breeding and Animal Ethology, Hugo Kołłątaj University of Agriculture in Krakow, ave. A.Mickiewicza 21, 31-120 Kraków, Poland; andrzej.weglarz@urk.edu.pl

**Keywords:** oxidative stress biomarkers, redox homeostasis, livestock, microbiome, Nrf2/NF-κB signaling, spatial management

## Abstract

Oxidative stress (OS) is a central regulator of health and productivity in livestock, emerging from complex interactions between dietary inputs, microbiome composition, environmental stressors, and host metabolism. This narrative review synthesizes current knowledge on OS in cattle, pigs, sheep, and poultry, emphasizing mechanistic pathways, tissue-specific responses, and translational applications. We highlight the central role of redox–inflammatory signaling hubs, including nuclear factor kappa B (NF-κB), nuclear factor erythroid 2–related factor 2 (Nrf2)/Kelch-like ECH-associated protein 1 (Keap1), and inflammasomes, as integrators of metabolic and immune stress. Microbiome–metabolome interactions modulate systemic oxidative responses, influencing liver, mammary gland, gastrointestinal tract, adipose tissue, and reproductive tissues. Oxidative stress-related biochemical and molecular alterations are captured by a range of biomarkers, such as malondialdehyde (MDA), Total Antioxidant Capacity (TOAC), gluthatione peroxidase (GPx), superoxide dismutase (SOD), paraoxonase-1 (PON1), cytokines, and gene expression profiles, measurable in blood, milk, saliva, and tissues. Integrating these markers enables precision diagnostics, early disease detection, and evidence-based nutritional interventions. Furthermore, computational modeling and spatial–socioeconomic perspectives offer novel approaches to translate molecular redox insights into practical livestock management strategies. By framing OS as a regulated, context-dependent process rather than a simple imbalance of reactive oxygen species, this review advances a conceptual, cross-species framework for understanding, monitoring, and mitigating oxidative stress in livestock. This integrative perspective provides a foundation for targeted antioxidant strategies and sustainable production practices, bridging molecular mechanisms with practical applications in animal health and productivity.

## 1. Introduction

Oxidative stress (OS), defined as an imbalance between the production of reactive oxygen and nitrogen species (ROS/RNS) and the antioxidant defense capacity of the organism, is increasingly recognized as a central molecular mechanism underlying immune dysfunction, metabolic disorders, impaired reproductive performance, and reduced productivity in livestock [1,2,3,4,5]. While ROS are essential signaling molecules involved in physiological processes such as cell proliferation, immune defense, and metabolic regulation, their excessive or dysregulated generation leads to oxidative damage of lipids, proteins, and nucleic acids, ultimately compromising cellular and tissue function [6,7,8,9,10].

In farm animals, oxidative stress is particularly relevant during physiologically demanding periods characterized by rapid metabolic shifts and increased energy requirements. The perinatal and periparturient periods represent vulnerable biological windows during which animals undergo profound endocrine, metabolic, and immunological adaptations to support fetal development, parturition, and the onset of lactation [11,12,13,14,15]. In dairy cows, sows, sheep, and other production species, late gestation and early lactation are associated with intensified mitochondrial activity, elevated lipid mobilization, negative energy balance, and increased oxygen consumption [16,17,18,19,20,21].

These processes collectively enhance ROS generation while simultaneously depleting antioxidant reserves, thereby predisposing animals to sustained oxidative stress and inflammatory dysregulation [12,22,23,24,25].

Importantly, oxidative stress should be viewed as a regulated and context-dependent biological process rather than a simple imbalance between ROS production and antioxidant defenses. Its integration with immune and metabolic signaling is primarily mediated by redox-sensitive transcription factors pathways, particularly nuclear factor erythroid 2–related factor 2 (Nrf2) and nuclear factor kappa B (NF-κB), which are discussed in detail in Section 2 [26,27,28,29,30,31,32,33,34,35,36].

Under physiological conditions, activation of Nrf2 promotes the transcription of antioxidant and cytoprotective genes encoding enzymes such as superoxide dismutase (SOD), glutathione peroxidase (GPx), catalase (CAT), and phase II detoxification enzymes [17,32,33]. In contrast, excessive or chronic activation of NF-κB drives pro-inflammatory cytokine production and further amplifies oxidative stress through immune cell activation and mitochondrial dysfunction [34,35,36,37]. Dietary composition represents one of the most powerful modulators of redox homeostasis in livestock. High-energy, high-concentrate diets, commonly used to support production demands, can exacerbate oxidative stress by promoting ruminal acidosis, microbial dysbiosis, and systemic inflammation [38,39,40,41,42,43]. Conversely, diets enriched with trace minerals, vitamins, functional amino acids, and bioactive compounds can enhance endogenous antioxidant capacity and modulate redox-sensitive signaling pathways [38,44,45,46,47]. Increasing attention has therefore been directed toward the concept of nutritional redox programming, whereby dietary interventions influence redox balance not only through direct antioxidant effects but also via transcriptional, metabolic, and epigenetic mechanisms [48,49,50,51,52].

In parallel, the role of the gut microbiome as a key regulator of oxidative stress has emerged as a critical area of research. Microbiota-derived metabolites, endotoxins, and signaling molecules interact with host immune and metabolic pathways, shaping systemic redox status [19,53,54,55]. Dysbiosis-induced increases in lipopolysaccharide (LPS) translocation can activate NF-κB signaling and inflammasome pathways, leading to enhanced ROS production and suppression of Nrf2-mediated antioxidant responses [31,34,35,36,37]. Conversely, beneficial microbial taxa and their metabolites may support redox homeostasis by reinforcing intestinal barrier integrity, modulating immune activation, and contributing to antioxidant capacity [56,57,58,59,60]. These complex interactions position the microbiome as a central intermediary linking diet to host redox biology [61,62,63,64,65,66].

**Figure 1:**
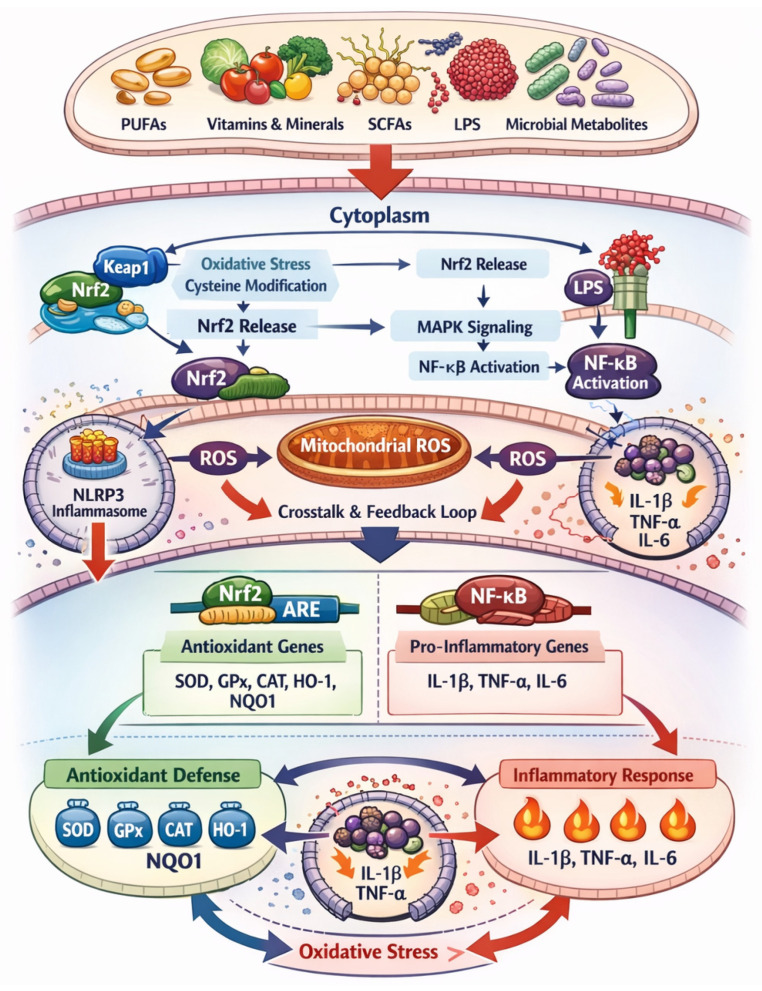
Integrated framework of oxidative stress biomarkers across biological matrices in livestock.

The integration of dietary inputs, microbiome-derived signals, and host redox-sensitive pathways is schematically summarized in (Figure 1), which illustrates the diet–microbiome–redox axis governing oxidative stress and inflammation in livestock. This conceptual framework highlights how nutritional strategies and microbial ecology converge on Nrf2/Keap1 and NF-κB signaling to determine tissue-specific oxidative and inflammatory outcomes [30,31,36,40]. Given the multifactorial nature of oxidative stress, reliable biomarkers are essential for assessing redox status, predicting disease risk, and guiding targeted interventions [67,68,69]. However, oxidative stress cannot be adequately captured by single biomarkers measured in isolation. Instead, it reflects the combined effects of tissue origin, physiological stage, dietary background, and regulatory signaling. This recognition has driven a shift toward integrated biomarker frameworks encompassing oxidative damage products, antioxidant defenses, and inflammation-related mediators measured across diverse biological matrices [70,71,72].

In this context, the present review aims to synthesize current knowledge on oxidative stress biomarkers in farm animals within an integrated diet–microbiome–redox–biomarker framework. By linking molecular mechanisms to measurable biomarker outputs and translational applications in animal production, this review seeks to provide a coherent foundation for precision nutrition, precision diagnostics, and sustainable livestock management strategies [72,73,74,75].

## 2. Molecular Pathways Integrating Oxidative Stress and Inflammation

Oxidative stress and inflammation are tightly interconnected biological processes that collectively determine immune competence, metabolic adaptation, and disease susceptibility in livestock species [76,77]. Rather than representing independent phenomena, redox imbalance and inflammatory activation are integrated through a network of redox-sensitive signaling pathways, among which nuclear factor erythroid 2–related factor 2 (Nrf2) and nuclear factor kappa B (NF-κB) constitute central and functionally antagonistic regulators [30,31,36,40].

### 2.1. The Keap1–Nrf2 Antioxidant Signaling Axis

Nuclear factor erythroid 2–related factor 2 acts as a master transcriptional regulator of cellular antioxidant and cytoprotective responses [30,31,36,40]. Under basal conditions, Nrf2 is retained in the cytoplasm through its interaction with Kelch-like ECH-associated protein 1 (Keap1), which promotes ubiquitination and proteasomal degradation of Nrf2 [36,40]. It also acts as a redox sensor via highly reactive cysteine residues that undergo oxidative or electrophilic modification under conditions of increased reactive oxygen species (ROS) production [30,31,32,33,34,35,36].

Oxidative modification of Keap1 disrupts the Keap1–Nrf2 complex, allowing Nrf2 stabilization and nuclear translocation. In the nucleus, Nrf2 heterodimerizes with small Maf proteins and binds to antioxidant response elements (AREs) located in promoter regions of target genes. This induces transcription of a wide range of antioxidant and detoxification enzymes, including superoxide dismutases (SOD), glutathione peroxidase (GPx), catalase (CAT), heme oxygenase-1 (HO-1), NAD(P)H:quinone oxidoreductase 1 (NQO1), and enzymes involved in glutathione synthesis and regeneration [17,36,40,78,79,80].

Beyond classical antioxidant defense, Nrf2 regulates genes associated with mitochondrial function, xenobiotic metabolism, iron homeostasis, and maintenance of redox buffering capacity [33]. In livestock species exposed to metabolic and inflammatory challenges—particularly during the periparturient transition—efficient activation of the Keap1–Nrf2 pathway is critical for maintaining redox homeostasis and limiting oxidative tissue damage [30,31,36,40].

### 2.2. NF-κB as a Central Mediator of Inflammatory–Redox Crosstalk

High-concentrate feeding promotes ruminal acidosis and endotoxin release, leading to LPS translocation and NF-κB activation [30,31,36,40].

Nuclear factor kappa B is a key transcription factor governing inflammatory and immune responses It is activated by a broad range of stimuli relevant to intensive livestock production systems, including lipopolysaccharide (LPS), pro-inflammatory cytokines, metabolic overload, and oxidative stress itself [31,81,82,83,84,85,86,87,88].

In ruminants fed high-concentrate diets, subacute ruminal acidosis promotes microbial lysis and LPS release. Disruption of epithelial barrier integrity facilitates LPS translocation into systemic circulation, where it activates Toll-like receptor 4 (TLR4), triggering downstream mitogen-activated protein kinase (MAPK) cascades and inhibitor of κB kinase (IKK) activation [6,30,54]. Inhibitor of κB kinase-mediated phosphorylation results in degradation of inhibitor of κB (IκB), enabling NF-κB nuclear translocation and transcriptional activation of pro-inflammatory genes [36,40,89].

Activated NF-κB induces expression of tumor necrosis factor-α (TNF-α), interleukin-1β (IL-1β), interleukin-6 (IL-6), chemokines, and adhesion molecules, thereby amplifying immune activation and promoting additional ROS production via NADPH oxidases and mitochondrial perturbation [33,36,40]. Importantly, ROS also function as secondary messengers that further enhance NF-κB activation, creating a self-sustaining inflammatory–oxidative feedback loop [90,91,92,93,94,95,96,97].

### 2.3. Mechanisms of NF-κB-Mediated Suppression of Nrf2 Signaling

Although Nrf2 and NF-κB may be transiently co-activated during acute stress, chronic or excessive NF-κB activation can suppress Nrf2-mediated antioxidant responses through several mechanisms [36,40,98,99].

First, NF-κB and Nrf2 compete for shared transcriptional coactivators, particularly CREB-binding protein (CBP)/p300. Sustained NF-κB activation preferentially recruits CBP/p300 to pro-inflammatory gene promoters, limiting coactivator availability for ARE-dependent transcription driven by Nrf2 [36,40,100,101].

Second, NF-κB-induced cytokine production enhances intracellular ROS generation via activation of NADPH oxidases and mitochondrial dysfunction. Persistent ROS elevation may promote Keap1-dependent ubiquitination and destabilization of Nrf2, impairing sustained antioxidant gene expression [33,36,40,101,102,103,104].

Third, IKK signaling has been shown to directly influence Nrf2 stability and transcriptional activity through phosphorylation-dependent mechanisms, while chronic inflammatory signaling may induce epigenetic modifications that reduce accessibility of ARE-containing promoters [30].

Collectively, these mechanisms shift the transcriptional balance toward a pro-inflammatory, pro-oxidant phenotype characterized by decreased antioxidant enzyme expression, enhanced lipid peroxidation, protein carbonyl formation, and mitochondrial dysfunction [3,32,80]. Such dysregulation is particularly relevant in high-producing dairy cows and rapidly growing monogastric species subjected to sustained metabolic and dietary stress [38,48,54].

### 2.4. Integration Within the Diet–Microbiome–Redox Axis

The Nrf2–NF-κB axis operates within a broader diet–microbiome–host interaction network. Nutritional composition influences redox signaling both directly, through provision of antioxidant micronutrients and bioactive compounds, and indirectly, via modulation of microbial ecology and metabolite production [36,40,101,102,103,104,105,106,107,108,109,110,111].

Diet-induced dysbiosis enhances endotoxin load and systemic inflammatory tone, favoring NF-κB activation. Conversely, targeted supplementation with selenium, vitamin E, B-complex vitamins, functional amino acids, polyphenols, and yeast-derived bioactive compounds may enhance Nrf2 activation, increase antioxidant enzyme activity, and attenuate inflammatory signaling [36,40,102,103,104,105,106,107,108,109,110,111].

Therefore, oxidative stress in livestock should be conceptualized as a dynamically regulated process emerging from interactions among dietary composition, microbial homeostasis, mitochondrial metabolism, and redox-sensitive transcriptional networks. A mechanistic understanding of Nrf2–NF-κB crosstalk provides a rational basis for biomarker selection and precision nutritional strategies aimed at improving redox resilience in modern production systems [36,40,102,103,104,105,106,107,108,109,110,111], (Figure 1).

This schematic summarizes the molecular mechanisms linking dietary factors, microbiome-derived metabolites, and redox-sensitive signaling pathways during metabolically demanding periods in livestock. Dietary components (PUFAs, vitamins, trace minerals, amino acids, plant-derived bioactives) and microbial metabolites modulate Keap1–Nrf2 and TLR4–MAPK–NF-κB signaling. Oxidative stress triggers cysteine modification of Keap1, allowing Nrf2 nuclear translocation and activation of ARE-dependent genes encoding antioxidant enzymes (SOD, GPx, CAT, HO-1, NQO1). LPS and other microbial signals activate NF-κB via MAPK pathways, increasing inflammatory cytokines (IL-1β, TNF-α, IL-6) and mitochondrial ROS production. ROS and NF-κB signaling promote inflammasome activation (e.g., NLRP3), creating redox–inflammatory feedback loops that can suppress Nrf2 if chronic. Functional nutritional strategies restore redox homeostasis by reactivating Nrf2, enhancing antioxidant capacity, modulating gut microbiota, and suppressing excessive NF-κB activation, thereby improving immune competence and production outcomes. Arrows indicate directionality of signaling interactions and biomarker responses, linking dietary factors and microbial dysbiosis to inflammatory activation and downstream oxidative damage.

## 3. Maternal Nutritional Programming and Epigenetic Regulation of Oxidative Stress in Offspring

Maternal nutrition during gestation and lactation has long-term consequences on offspring health through epigenetic modifications. Long-chain polyunsaturated fatty acids (PUFAs), particularly n-3 PUFAs, modulate redox balance and immune development [32,39,76,98,106]. Maternal fatty acid intake influences deoxyribonucleic acid (DNA) methylation patterns and transcriptional activity of genes related to lipid metabolism, mitochondrial function, and antioxidant defenses in offspring, supporting translational approaches to improving livestock resilience [5,32,33,79].

Increasing evidence indicates that oxidative stress plays a central role in mediating these programming effects by influencing epigenetic regulation, mitochondrial function, and immune development in the developing fetus and neonate [33,87,103]. In livestock species, where reproductive efficiency, neonatal survival, and growth performance are critical determinants of production outcomes, understanding the nutritional regulation of redox balance during early life stages has important translational implications [25,76,83].

Epigenetic mechanisms, including DNA methylation, histone modifications, and non-coding ribonucleic acid (RNA) regulation, provide a molecular interface through which maternal dietary signals are integrated into long-term changes in gene expression without altering the underlying DNA sequence. These epigenetic marks are particularly sensitive during critical windows of development, such as early gestation, placentation, and early postnatal life, when rapid cell proliferation and tissue differentiation occur. Oxidative stress can directly influence epigenetic processes by modifying the availability of methyl donors, altering chromatin structure, and regulating the activity of epigenetic enzymes, thereby linking redox status to long-term transcriptional reprogramming [34,45,46,47].

Among nutritional factors, long-chain polyunsaturated fatty acids (PUFAs), particularly n-3 PUFAs, have received considerable attention for their role in modulating oxidative stress, inflammation, and immune development. Polyunsaturated fatty acids (n-3 PUFAs) influence membrane composition, mitochondrial function, and redox-sensitive signaling pathways, including Nrf2 and NF-κB. Maternal supplementation with n-3 PUFAs during gestation has been shown to reduce inflammatory tone and oxidative stress in dams, while simultaneously shaping antioxidant capacity and immune competence in offspring [5,32,36,39,40]. However, the effects of PUFA supplementation are highly dose- and timing-dependent, as excessive unsaturated fatty acid intake may increase lipid peroxidation and oxidative burden if not accompanied by adequate antioxidant support [48,103,106].

Maternal fatty acid intake has been demonstrated to influence DNA methylation patterns and transcriptional activity of genes involved in lipid metabolism, mitochondrial biogenesis, and antioxidant defense in offspring tissues. Studies in pigs, sheep, and cattle have revealed that prenatal nutritional modulation alters the expression of key genes regulating mitochondrial oxidative phosphorylation, fatty acid oxidation, and glutathione metabolism, thereby affecting redox homeostasis beyond the immediate postnatal period These epigenetic adaptations may partially explain observed associations between maternal diet, offspring growth performance, immune responsiveness, and susceptibility to metabolic and inflammatory disorders later in life [4,45,55,68,86].

Mitochondria represent a critical nexus between nutritional programming, oxidative stress, and epigenetic regulation. As the primary source of cellular ROS, mitochondrial function is highly sensitive to both maternal nutrient availability and oxidative environment during development. Alterations in mitochondrial number, morphology, and respiratory efficiency induced by maternal diet can persist into postnatal life, influencing energy metabolism and redox balance. Epigenetic regulation of mitochondrial-related nuclear genes, including those encoding antioxidant enzymes and electron transport chain components, further reinforces the long-term impact of early nutritional exposures [33].

Beyond fatty acids, maternal intake of trace minerals, vitamins, and functional amino acids also contributes to epigenetic regulation of oxidative stress. Selenium, zinc, and copper are essential cofactors for antioxidant enzymes, while vitamins such as vitamin E and B-complex vitamins participate in redox regulation and one-carbon metabolism. Deficiencies or imbalances in these nutrients during gestation can exacerbate oxidative stress and disrupt epigenetic programming, whereas targeted supplementation may enhance redox resilience and immune competence in offspring [38,44,46].

The role of the maternal microbiome in nutritional programming is an emerging area of interest. Diet-induced alterations in maternal gut microbiota composition influence microbial metabolite profiles, immune signaling, and oxidative status, which can indirectly affect fetal development through placental signaling and nutrient transfer. Although this field is still evolving in livestock research, parallels from other animal models suggest that microbiome-mediated modulation of redox balance may represent an additional layer of epigenetic regulation linking maternal diet to offspring health [48,103,106].

These interconnected mechanisms are integrated within the conceptual framework presented in (Figure 1), which illustrates how maternal dietary inputs and microbiome-derived signals converge on redox-sensitive pathways to influence epigenetic programming and long-term physiological outcomes [7,32,39]. This framework underscores the translational potential of targeting maternal nutrition to modulate oxidative stress and improve resilience, health, and productivity across generations in livestock systems.

From an applied perspective, nutritional programming of oxidative stress offers promising opportunities for intervention at the earliest stages of life. By optimizing maternal diets to support balanced redox signaling and epigenetic stability, it may be possible to enhance offspring robustness, reduce disease susceptibility, and improve lifetime performance. Such strategies align with the broader goals of precision nutrition and sustainable livestock production, emphasizing proactive rather than reactive management of oxidative stress [25,76,83].

## 4. Microbiome–Metabolome–Redox Interactions

The gut, mammary, and uterine microbiomes are increasingly recognized as integral modulators of oxidative status and immune function in livestock, acting as dynamic interfaces between diet, host metabolism, and inflammatory signaling [96,102,109]. These microbial ecosystems not only influence nutrient utilization and barrier integrity but also shape redox homeostasis through the production of metabolites, regulation of host enzymatic pathways, and modulation of immune responses [27,38,45,77]. Disruptions in microbiome composition—commonly referred to as dysbiosis—can therefore amplify oxidative stress and contribute to the pathogenesis of production-limiting diseases [77,78,79,80,81,82,83].

In the mammary gland, mastitis represents a well-characterized model illustrating the link between microbial dysbiosis, oxidative stress, and inflammation. High-throughput sequencing studies have revealed that mastitis-affected cows exhibit distinct shifts in milk microbiota, with increased relative abundance of genera such as *Streptococcus*, *Acinetobacter*, and *Romboutsia* [102]. These microbial changes are accompanied by profound alterations in the milk metabolome, including elevated levels of purine metabolites and oxidative stress–related compounds. Notably, increased xanthine oxidase activity has been implicated as a key source of reactive oxygen species (ROS) in mastitic mammary tissue, contributing to lipid peroxidation, epithelial damage, and impaired milk synthesis [5,32,80]. These findings suggest that microbial-driven metabolic reprogramming plays a direct role in amplifying local oxidative stress during intramammary infection [32,44,89].

Similar host–microbiome–redox interactions have been described in the reproductive tract, particularly during uterine inflammatory disorders such as metritis and endometritis. Pathogenic bacteria, including *Fusobacterium necrophorum*, have been shown to exacerbate uterine oxidative stress by stimulating innate immune responses and promoting excessive ROS generation [94,95,96,97,102]. Inflammatory activation of uterine epithelial and immune cells leads to increased expression of NADPH oxidases and mitochondrial dysfunction, further reinforcing oxidative damage to endometrial tissues. This oxidative environment not only impairs uterine repair and involution but may also compromise subsequent fertility by disrupting hormonal signaling and embryo–maternal communication [33,82].

The gastrointestinal tract represents a central hub in the diet–microbiome–redox axis, particularly in ruminants. Dietary-induced shifts in rumen microbial communities influence the production of short-chain fatty acids, endotoxins, and redox-active metabolites [37,47,53]. High-concentrate diets and subacute ruminal acidosis promote microbial lysis and lipopolysaccharide (LPS) release, which can translocate across a compromised epithelial barrier and trigger systemic inflammatory and oxidative responses [31]. LPS-mediated activation of Toll-like receptor signaling enhances NF-κB-dependent inflammation while suppressing Nrf2-driven antioxidant defenses, thereby creating a pro-oxidant systemic milieu that affects distant tissues, including the mammary gland and uterus (Figure 1), [31,36,40].

Beyond direct pathogen effects, commensal microbes and their metabolites also exert protective roles in maintaining redox balance. Short-chain fatty acids, microbial antioxidants, and indole derivatives have been shown to support epithelial integrity, modulate immune tolerance, and activate antioxidant signaling pathways [53,54,55]. Disruption of these beneficial microbial functions during periods of metabolic stress, such as early lactation, may therefore predispose animals to oxidative injury and inflammatory disease [71,89]. This dual role of the microbiome—as both a source of oxidative stress and a regulator of antioxidant resilience—underscores the complexity of host–microbiome interactions [1,2,3,4,5,6,7,19,30].

Collectively, these findings highlight host–microbiome–metabolome crosstalk as a critical determinant of redox homeostasis in livestock, integrating dietary inputs, microbial ecology, immune activation, and oxidative signaling [30]. As schematically illustrated in (Figure 1), microbial dysbiosis acts as a key upstream driver linking nutritional stress to redox imbalance and tissue dysfunction [34,45,46,47]. From a translational perspective, targeting the microbiome through precision feeding, probiotics, prebiotics, and microbiota-modulating strategies offers a promising avenue to mitigate oxidative stress, improve animal health, and enhance production efficiency [53,54,55].

## 5. Biomarkers of Oxidative Stress Across Biological Matrices

**Figure 2:**
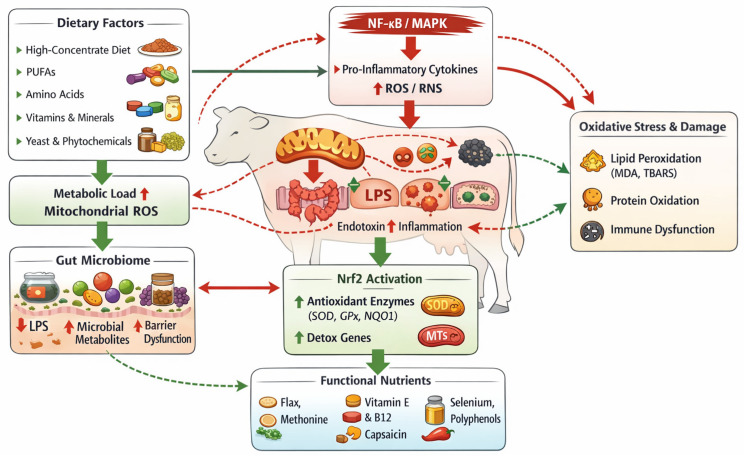
Diet–Microbiome–Nrf2/NF-κB Axis in the Regulation of Oxidative Stress in Periparturient Livestock.

Reliable and biologically meaningful biomarkers are essential for the assessment, interpretation, and management of oxidative stress in livestock systems, providing mechanistic insight into redox dynamics and guiding targeted interventions [34,45,46,47]. Given the tissue-specific and dynamic nature of redox regulation, no single biomarker can fully capture oxidative status. Instead, oxidative stress is best evaluated using integrated panels of biomarkers that reflect oxidative damage, antioxidant defense capacity, and regulatory redox signaling across multiple biological matrices [34,45,46,47]. As illustrated in (Figure 2), these biomarkers originate from distinct tissues and are detectable in diverse matrices, providing complementary insights into systemic and local redox homeostasis [34,45,46,47].

Among oxidative damage indicators, lipid peroxidation products remain the most extensively applied biomarkers in farm animal research. Malondialdehyde (MDA), thiobarbituric acid-reactive substances (TBARS), and F2-isoprostanes reflect reactive oxygen species (ROS)-induced peroxidation of polyunsaturated fatty acids and are particularly sensitive to metabolic and inflammatory stress [5,32,80]. In ruminants, elevated plasma and serum concentrations of these markers are consistently observed during the periparturient and early lactation periods, when negative energy balance, immune activation, and mitochondrial overload converge to increase oxidative burden [38,44]. F2-isoprostanes are considered gold-standard biomarkers due to their chemical stability, specificity, and relative independence from dietary lipid composition, making them especially robust indicators of oxidative stress in periparturient dairy cows (Figure 2) [51,52,53,54,55,56,57,58].

Protein oxidation markers, particularly protein carbonyls, provide complementary information by capturing irreversible oxidative modifications of structural and enzymatic proteins [17]. These markers are less influenced by short-term dietary fluctuations and are therefore well suited for assessing chronic oxidative stress and sustained inflammatory states. Elevated protein carbonyl levels have been reported in cattle, pigs, and sheep exposed to heat stress, lipopolysaccharide (LPS) challenge, or intensive production conditions, supporting their utility as integrative indicators of long-term redox imbalance [17,31]. In contrast, biomarkers of nucleic acid oxidation, such as oxidized purines and related metabolites, are less frequently employed in livestock but represent an emerging area of interest, particularly in studies linking oxidative stress to mitochondrial dysfunction and immune regulation [33].

In parallel with damage-related markers, antioxidant defense biomarkers provide insight into the host’s adaptive capacity to counteract oxidative insults [17,18,19,20,21]. Enzymatic antioxidants, including superoxide dismutase (SOD), glutathione peroxidase (GPx), and catalase (CAT), constitute the primary cellular defense against ROS accumulation and are tightly regulated by redox-sensitive transcriptional pathways [17,18,19,20]. Measurement of these enzymes in plasma, erythrocytes, milk, and tissue samples has revealed pronounced changes in response to nutritional interventions, physiological stage, and disease status. For example, increased GPx activity following organic selenium supplementation reflects enhanced endogenous antioxidant protection rather than direct radical scavenging, highlighting the mechanistic relevance of these biomarkers [12,17,18,19,20,21,22,23,24,25] (Figure 2).

Global antioxidant indices, such as total antioxidant capacity (T-AOC) or total antioxidant status (TAS), integrate the combined effects of enzymatic antioxidants, non-enzymatic micronutrients, and redox-active metabolites [47,48,53]. While these indices lack molecular specificity, they offer a valuable system-level readout of redox homeostasis and are particularly useful for monitoring responses to dietary supplementation strategies. Importantly, interpretation of T-AOC and TAS requires careful consideration of physiological context and matrix-specific reference values, as contributions from uric acid, bilirubin, and microbial metabolites may differ across species and tissues [19,20,47,48].

The biological matrix used for biomarker assessment critically determines translational applicability. Blood-derived matrices (plasma and serum) remain the most widely used due to their accessibility and systemic relevance [79,90,95]. However, increasing attention is being directed toward non-invasive matrices, such as milk, saliva, ruminal fluid, urine, and colostrum, which enable repeated sampling and improved animal welfare [4,26,98,102]. Milk and colostrum biomarkers provide localized insight into mammary redox status and immune activation, particularly in the context of mastitis and early lactation [42,44]. Ruminal fluid reflects diet–microbiome–redox interactions, capturing oxidative processes linked to fermentation and microbial dysbiosis [2,8,59].

Saliva has emerged as a particularly promising matrix for oxidative stress monitoring, especially in pigs and small ruminants [35,59,67,69,71]. Salivary biomarkers, including lipid peroxidation products and antioxidant enzymes, correlate well with systemic oxidative status and respond sensitively to environmental, nutritional, and inflammatory stressors. Their non-invasive nature supports longitudinal monitoring at both individual and herd levels, enhancing their translational value for on-farm diagnostics [35,59,67,69,71].

Collectively, the framework presented in Figure 2 emphasizes that oxidative stress biomarkers should be interpreted within an integrated tissue–matrix context rather than as isolated measurements [17,33]. Combining damage markers, antioxidant defenses, and matrix-specific readouts enables a more nuanced understanding of redox dynamics and supports the development of precision nutrition and management strategies aimed at enhancing redox resilience in livestock production systems (Table 1), [34,45,46,47].

## 6. Functional Nutritional Strategies to Mitigate Oxidative Stress

Functional nutrition has emerged as a central pillar in the management of oxidative stress in livestock, extending well beyond the classical concept of direct antioxidant supplementation. Rather than acting solely as increased reactive oxygen species (ROS) scavengers, functional dietary components exert pleiotropic effects on redox-sensitive signaling pathways, immune regulation, mitochondrial metabolism, and gut microbial ecology. This multi-target mode of action is particularly relevant during physiologically demanding periods, such as late gestation, early lactation, rapid growth, and environmental stress exposure, when redox homeostasis is most vulnerable (Figure 1 and Figure 2) [4,42,43].

Amino acids play a critical role in maintaining intracellular redox balance by serving as precursors for antioxidant molecules and by modulating metabolic signaling pathways. Methionine, through its involvement in the transsulfuration pathway, supports glutathione synthesis and methyl-group metabolism, thereby linking antioxidant defense with epigenetic regulation. Lysine supplementation has been shown to improve immune competence and reduce oxidative damage, particularly under conditions of metabolic stress, by supporting protein turnover and mitochondrial efficiency [44]. These effects underscore the importance of amino acid adequacy not only for growth and production but also for redox resilience.

Trace minerals such as selenium, zinc, and copper are indispensable cofactors for antioxidant enzymes, including glutathione peroxidase (GPx), superoxide dismutase (SOD), and catalase (CAT). Organic and rumen-protected forms of selenium have demonstrated superior bioavailability and efficacy in activating Nrf2-dependent antioxidant pathways compared with inorganic sources [17]. Zinc and copper further contribute to immune modulation and maintenance of epithelial barrier integrity, thereby indirectly reducing oxidative and inflammatory load originating from the gastrointestinal tract (Figure 1 and Figure 2) [110].

Fat-soluble and water-soluble vitamins represent another key component of functional nutritional strategies. Vitamin E remains a cornerstone lipid-phase antioxidant, protecting cellular membranes from lipid peroxidation, particularly in high-producing dairy cows. Beyond its classical antioxidant role, vitamin E modulates immune cell signaling and inflammatory gene expression. Vitamin B12, through its involvement in one-carbon metabolism and mitochondrial function, has gained increasing attention for its indirect effects on redox balance and energy metabolism, especially in ruminants with high metabolic demands [18,38,44].

Microbiome-targeted nutritional interventions, including yeast cultures and probiotics, provide an additional layer of redox modulation by stabilizing gut microbial composition and enhancing intestinal barrier function. Yeast-based additives have been shown to reduce endotoxin translocation, attenuate NF-κB activation, and stimulate endogenous antioxidant systems in both ruminants and monogastric species [53,59,81]. By limiting lipopolysaccharide (LPS)-induced inflammatory signaling, these interventions reduce secondary ROS production and support systemic redox homeostasis (Figure 1 and Figure 2, Table 1) [34,45,46,47].

Plant-derived bioactive compounds, such as polyphenols and capsaicin, have attracted growing interest due to their ability to activate redox-sensitive transcriptional programs rather than merely neutralizing ROS. Polyphenols interact with Keap1–Nrf2 signaling, promoting the expression of phase II detoxifying and antioxidant enzymes, while capsaicin improves mitochondrial efficiency and reduces oxidative damage in metabolically active tissues [36,40,92,106]. These compounds exemplify the shift from antioxidant replacement toward redox signaling modulation.

Evidence from dietary intervention studies highlights the translational relevance of functional nutrition. Flax meal supplementation in dairy cows increased Nrf2, mitochondrial RNA, (mRNA) abundance in mammary tissue and significantly reduced thiobarbituric acid-reactive substances (TBARS), demonstrating transcriptional reprogramming of antioxidant defenses rather than a transient antioxidant effect [32,111]. Similarly, yeast-based additives improved gut microbiota composition, enhanced total antioxidant capacity (T-AOC), and reduced systemic oxidative stress markers, illustrating the interconnectedness of diet, microbiome, and redox regulation (Figure 1 and Figure 2) [45,46,47].

Importantly, the effectiveness of functional nutrition strategies depends on precise formulation, timing, and physiological context. Excessive or unbalanced antioxidant supplementation may blunt physiological redox signaling required for immune activation and metabolic adaptation. Therefore, precision nutrition guided by validated oxidative stress biomarkers (Figure 2) is essential to optimize redox homeostasis without disrupting redox-dependent cellular communication. This biomarker-driven approach represents a key translational bridge between molecular redox biology and practical livestock nutrition, supporting sustainable improvements in animal health, welfare, and productivity [67,68,69,70,71,72], (Figure 2).

Figure 2 provides a schematic representation of the integrated molecular mechanisms linking dietary factors, microbiome composition, and redox-sensitive signaling pathways in the regulation of oxidative stress in livestock during metabolically demanding periods, such as periparturient and perinatal phases. Nutritional inputs-including high-concentrate diets, polyunsaturated fatty acids (PUFAs), amino acids, trace minerals, vitamins, yeast cultures, and plant-derived bioactive compounds-directly influence metabolic load and mitochondrial ROS production [39]. Diet-induced alterations in ruminal and intestinal microbiota modulate endotoxin (lipopolysaccharide, LPS) release, microbial metabolites (e.g., xanthine, quinic acid), and barrier integrity, thereby shaping systemic inflammatory tone [31,47,51,53].

Microbial-derived signals and metabolic stress activate pro-inflammatory pathways, predominantly NF-κB and MAPK, leading to increased cytokine expression and enhanced production of reactive oxygen and nitrogen species (ROS/RNS) [17,36,40]. Concurrently, chronic inflammatory signaling suppresses Nrf2 (NFE2L2)-dependent antioxidant responses, resulting in downregulation of cytoprotective genes encoding antioxidant enzymes (SOD, GPx, CAT, NQO1, MT1A/MT1E) and phase II detoxification systems [19,34,38,44]. This transcriptional imbalance promotes lipid peroxidation (elevated MDA, TBARS, F2-isoprostanes), protein oxidation, and impaired immune cell function, collectively compromising tissue homeostasis and production performance [12].

**Figure 3:**
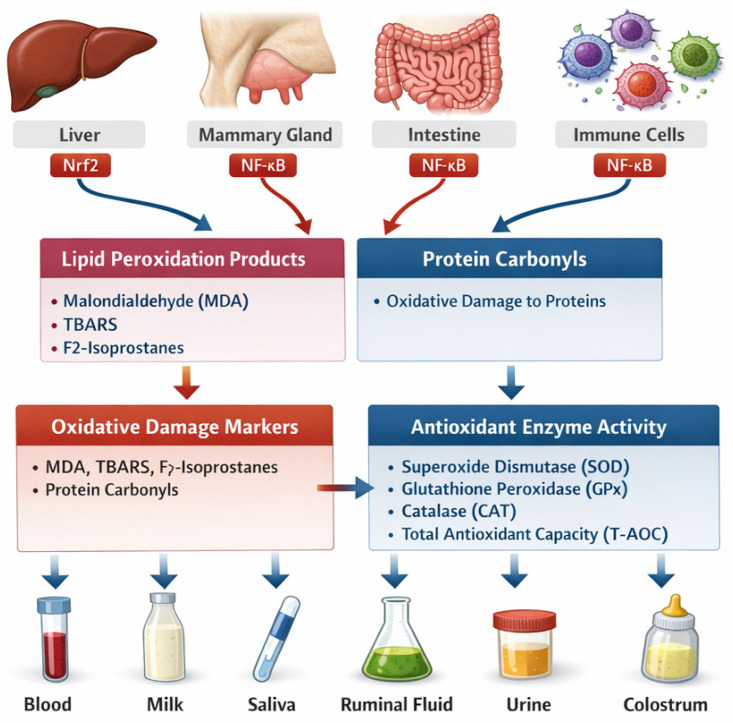
Overview of Integrated Tissue–Biomarker–Matrix Framework for the Assessment of Oxidative Stress in Livestock. Tissue sources (liver, mammary gland, intestine, immune cells) contribute to lipid peroxidation products (MDA, TBARS, F2-isoprostanes) and protein carbonyls. Antioxidant enzyme activity includes SOD, GPx, CAT, and T-AOC. Biomarkers are measured in blood, milk, saliva, ruminol fluid, urine and colostrum.

Functional nutritional strategies—including flax meal, methionine, selenium, vitamins E and B12, yeast-derived compounds, polyphenols, and capsaicin—restore redox homeostasis by reactivating Nrf2 signaling, enhancing endogenous antioxidant capacity, improving mitochondrial efficiency, and modulating gut microbiota composition [31,63,64]. This framework emphasizes oxidative stress as a dynamic, regulated process emerging from diet–microbiome–host interactions rather than a mere consequence of excessive ROS production, ultimately influencing disease susceptibility, immune competence, reproductive performance, and overall productivity in livestock systems (Figure 3).

Figure 3 schematically summarizes the relationships between oxidative stress biomarkers, their tissue-specific origins, regulatory redox pathways, and the biological matrices used for their quantification in livestock species. Oxidative damage is represented by lipid peroxidation markers—including malondialdehyde (MDA), thiobarbituric acid-reactive substances (TBARS), and F2-isoprostanes—as well as protein oxidation indicators such as protein carbonyls and selected nucleic acid damage markers [16,17,18,19,20,21]. Complementarily, antioxidant status is evaluated through enzymatic defenses, including superoxide dismutase (SOD), glutathione peroxidase (GPx), catalase (CAT), and glutathione reductase, together with non-enzymatic components such as total antioxidant capacity (T-AOC/TAS), vitamins, and bioactive peptides [17,38,44].

These biomarkers originate from multiple metabolically and immunologically active tissues, including the liver (central to metabolic detoxification and lipid metabolism), mammary gland (milk synthesis and immune defense), rumen and intestine (microbiome–host interactions), adipose tissue (endocrine signaling), immune organs and circulating leukocytes, as well as placental and reproductive tissues during gestation [41,79,90,95]. Tissue-specific oxidative responses are modulated by metabolic load, inflammatory signaling, and redox-sensitive transcriptional regulation, particularly through the Nrf2 and NF-κB pathways, which integrate antioxidant defense and pro-inflammatory activation [2,36,40].

Biomarker detection is possible in diverse biological matrices—including blood (plasma, serum), milk, ruminal fluid, saliva, urine, and colostrum—supporting both invasive and non-invasive monitoring strategies across livestock species [4,17,26,98]. The figure underscores that appropriate biomarker and matrix selection must be adapted to the physiological stage (e.g., periparturient period, lactation, weaning) and specific disease context (e.g., mastitis, metritis, metabolic disorders) to ensure accurate characterization of oxidative status and to inform targeted nutritional or therapeutic interventions [42,44,102,107].

Overall, these biomarkers highlights that oxidative stress assessment in livestock should rely on coordinated interpretation of damage markers, antioxidant defenses, tissue origin, regulatory signaling pathways, and matrix-specific considerations, rather than on isolated biochemical measurements.

### 6.1. Computational and Informatics Approaches in Redox Biomarker Integration

The growing complexity of oxidative stress biology in livestock, encompassing diet–microbiome interactions, redox-sensitive signaling pathways, and multi-matrix biomarker datasets (Figure 1, Figure 2 and Figure 3), necessitates advanced informatics and computational approaches for meaningful integration and interpretation [34,44,45,46,47]. Traditional univariate analyses are insufficient to capture the nonlinear, multi-scale relationships that govern redox homeostasis across tissues, physiological stages, and production systems.

Applied informatics, mathematical modeling, and data-driven analytics provide powerful tools to translate high-dimensional oxidative stress data into actionable biological and management insights [45,46,47,48,49,50,51,52,53,54]. Multivariate statistics, machine learning algorithms, and network-based modeling enable the identification of biomarker signatures that reflect underlying redox–inflammatory states rather than isolated oxidative damage events. For example, integrating antioxidant enzyme activities, lipid peroxidation markers, inflammatory mediators, and microbiome-derived features allows the construction of predictive models for disease susceptibility, metabolic imbalance, and productivity outcomes [48,49,50,51,52].

From a systems biology perspective, computational frameworks facilitate the mapping of diet–microbiome–redox interactions into regulatory networks linking Nrf2/Keap1, NF-κB, mitochondrial metabolism, and immune signaling [36,40,51,53]. Such models can incorporate temporal dynamics, enabling longitudinal tracking of redox trajectories during critical physiological windows such as the periparturient period. Importantly, these approaches support the identification of early-warning biomarkers that precede clinical manifestation of disorders such as mastitis, metritis, or metabolic disease [36,40,102,107].

In the context of functional and precision nutrition, informatics-driven decision-support systems can optimize dietary interventions based on biomarker feedback [9,11,102]. By integrating oxidative stress biomarkers (Figure 2), nutritional inputs, and environmental variables, predictive algorithms may guide individualized or herd-level nutritional strategies that enhance redox resilience while avoiding disruption of physiological redox signaling. This aligns with the concept of redox-informed precision livestock farming, where nutritional and management decisions are supported by quantitative, model-based evidence [68].

The contribution of applied informatics and mathematical modeling thus represents a critical translational bridge between molecular redox biology and practical implementation in livestock production systems. By enabling scalable data integration, predictive analytics, and decision support, computational approaches enhance the translational impact of oxidative stress research and support sustainable, data-driven animal health management. This interdisciplinary framework directly reflects the growing role of informatics and systems-level analysis in modern redox biology and precision agriculture [34,45,46,47].

### 6.2. Socio-Economic and Spatial Dimensions of Redox-Informed Livestock Production

Oxidative stress in livestock, while rooted in molecular and cellular mechanisms, manifests at broader socio-economic and spatial scales that shape animal health, productivity, and sustainability of production systems [49,60,72]. The diet–microbiome–redox axis represents a central integrative framework linking nutritional inputs with host oxidative and inflammatory signaling (Figure 1). The biomarker-based assessment strategy summarized in (Figure 2 and Figure 3) provide a biological foundation for understanding how management practices, environmental conditions, and regional production systems influence oxidative stress burden in farm animals [47,68].

From a spatial management perspective, livestock production systems operate within heterogeneous agro-ecological landscapes characterized by differences in climate, feed availability, land use, and infrastructure. These spatial factors strongly influence nutritional strategies, exposure to environmental stressors (e.g., heat stress, housing density), and consequently oxidative stress levels. For instance, regions with intensive production systems and high-concentrate feeding are more prone to diet-induced redox imbalance, whereas extensive or pasture-based systems may face seasonal oxidative challenges related to nutrient variability and climatic extremes [70,71,73,78].

Socio-economic determinants further modulate oxidative stress outcomes through their impact on management decisions, resource allocation, and access to nutritional technologies [76]. Farm size, economic constraints, and market pressures often dictate the feasibility of implementing precision nutrition strategies, antioxidant supplementation, and biomarker-based monitoring programs. Integrating redox biology with socio-economic analysis enables identification of production contexts in which oxidative stress mitigation yields the greatest return on investment in terms of animal health, productivity, and welfare [75,76,83].

Spatially explicit approaches, including geographic information systems (GIS) and regional production modeling, offer valuable tools for linking oxidative stress risk with environmental and management variables [1,2,3,4]. By mapping biomarker-informed indicators of redox imbalance against climatic zones, feeding systems, and disease prevalence, it becomes possible to identify regional “hotspots” of oxidative stress vulnerability. Such analyses can support targeted interventions, optimized feed formulation strategies, and region-specific health management programs [8,35,56].

At the policy and industry level, redox-informed livestock management aligns with broader goals of sustainable agriculture, reduced antimicrobial usage, and improved animal welfare [61,62]. Biomarker-guided nutritional interventions have the potential to reduce disease incidence and production losses, contributing to economic resilience at both farm and regional scales. Moreover, integrating oxidative stress metrics into sustainability assessments may enhance evaluation frameworks used in agri-environmental policies and certification schemes [72,73,74,75].

In this context, the socio-economic and spatial perspective provides a critical translational layer linking molecular redox mechanisms with real-world livestock production systems. By embedding oxidative stress biology within regional, economic, and spatial frameworks, this interdisciplinary approach supports evidence-based decision-making, promotes sustainable intensification, and enhances the resilience of livestock systems across diverse geographic and socio-economic settings [34].

## 7. Biomarkers of Oxidative Stress in Farm Animals

Oxidative stress in farm animals is increasingly recognized as a dynamic, regulated biological process rather than a simple imbalance between reactive oxygen species (ROS) production and antioxidant availability [1,2,3,4,5,6,7]. As outlined in Figure 1, dietary composition, metabolic load, and microbiome-derived signals converge on redox-sensitive pathways—most notably the Nrf2/Keap1 and NF-κB signaling axes—thereby shaping tissue-specific oxidative and inflammatory responses [5,36,40]. The downstream molecular consequences of this redox dysregulation are captured through a diverse array of biomarkers, whose biological relevance depends on their tissue of origin and the matrix in which they are measured, as summarized in (Figure 2) [34,45,46,47].

### 7.1. Antioxidant Defense Biomarkers and Redox Capacity

In contrast to damage-related indicators, antioxidant biomarkers provide insight into the host’s adaptive response to oxidative stress. Enzymatic antioxidants, including superoxide dismutase (SOD), glutathione peroxidase (GPx), and catalase (CAT), represent the first line of defense against ROS accumulation and are tightly regulated by Nrf2-dependent transcriptional mechanisms (Figure 1), [17,36,40]. Dietary supplementation with organic selenium, rumen-protected amino acids, vitamins, yeast-derived products, and plant bioactives has been shown to enhance the activity of these antioxidant enzymes in blood, milk, and tissue samples of periparturient cows and growing pigs [17,41,56,64].

Non-enzymatic antioxidant capacity, commonly expressed as total antioxidant capacity (T-AOC or TAS), integrates the cumulative effects of endogenous antioxidants, dietary micronutrients, and microbial metabolites. Within the framework presented in (Figure 2), T-AOC serves as a system-level readout of redox homeostasis and has been demonstrated to respond sensitively to nutritional interventions, including capsaicin supplementation, combined antioxidant additives, and selenium-enriched yeast, across multiple livestock species [53,59,81].

### 7.2. Inflammation–Redox Crosstalk and Regulatory Biomarkers

A defining feature of oxidative stress in farm animals is its intimate connection with inflammatory signaling, forming a bidirectional inflammation–redox feedback loop (Figure 1). Pro-inflammatory stimuli, including lipopolysaccharide (LPS) derived from dysbiotic gut microbiota or high-concentrate feeding, activate NF-κB and inflammasome pathways, leading to enhanced ROS production and suppression of Nrf2-mediated antioxidant responses [1,2,3,4,36,40]. This reciprocal interaction between oxidative stress and inflammation has been extensively documented in periparturient dairy cows, where redox imbalance contributes to immune dysfunction and metabolic disorders [10,59,64]. Biomarkers reflecting this crosstalk—such as circulating cytokines, acute-phase proteins, malondialdehyde (MDA), paraoxonase-1 (PON1), and purine metabolites—have gained increasing attention in recent years as integrated indicators of inflammatory–oxidative status (Figure 2) [12].

At the molecular level, regulatory elements such as microRNAs further integrate oxidative and inflammatory signaling. For example, miR-223 has been shown to modulate NLRP3 inflammasome activity and Keap1 expression, thereby attenuating LPS-induced oxidative stress and inflammation in bovine mammary epithelial cells and mammary tissue models [30]. These findings underscore the importance of moving beyond single biomarkers toward regulatory and signaling-oriented indicators of redox status, incorporating pathway-level mediators (e.g., NF-κB, Nrf2, inflammasomes, and microRNAs) to better characterize redox–immune interactions in livestock (Table 1).

### 7.3. Tissue-Specific Origins of Oxidative Stress Biomarkers

As illustrated in (Figure 3), oxidative stress biomarkers originate from multiple tissues, each contributing distinct molecular signatures. The liver plays a central role in redox regulation through its involvement in lipid metabolism, detoxification, and antioxidant synthesis, making it a primary source of circulating oxidative stress markers in ruminants [2,35,59]. High-grain feeding and endotoxin translocation have been shown to provoke hepatic oxidative stress and activation of MAPK and Nrf2 signaling pathways in dairy cows [36,40].

The mammary gland represents a critical site of oxidative and inflammatory stress during early lactation, particularly in the context of mastitis and immune activation [4,42,43]. LPS challenge and mammary epithelial cell models demonstrate coordinated activation of inflammatory and oxidative pathways, further supporting the mammary gland as both a target and contributor to systemic redox imbalance [41,42,43,44].

In parallel, the rumen and intestinal tract, shaped by diet composition and microbiome dynamics (Figure 1 and Figure 2), act as both sources and modulators of oxidative stress through microbial metabolites, endotoxin release, and barrier integrity disruption [85,93]. Alterations in gut microbiota have been linked to changes in glutathione metabolism and systemic oxidative status in postpartum dairy cows [41,42,43], highlighting the gut–liver–mammary axis as a key integrative pathway in redox regulation.

Additional tissues, including adipose tissue, immune cells, and placental and reproductive tissues, contribute to systemic redox balance, especially during gestation and early life stages [44,54,56]. Studies in pigs and sheep have demonstrated that maternal oxidative stress can profoundly affect placental function, fetal development, and neonatal health, reinforcing the concept of oxidative stress as a whole-organism, yet tissue-specific phenomenon (Table 1).

### 7.4. Biological Matrices and Translational Relevance

The translational value of oxidative stress biomarkers depends strongly on the biological matrix used for their assessment (Figure 2). Blood-derived matrices (plasma and serum) remain the most common due to their accessibility and systemic relevance, particularly in transition dairy cows and animals exposed to metabolic or inflammatory challenges [32,44]. However, increasing emphasis is placed on non-invasive matrices, such as saliva, urine, milk, and colostrum, which allow repeated sampling and improved animal welfare. Salivary biomarkers, in particular, have demonstrated promising analytical performance and sensitivity to physiological and environmental stressors in pigs and sheep, supporting their inclusion in future redox monitoring strategies [45,46,47]. Milk and colostrum also reflect local mammary oxidative status and neonatal redox adaptation, providing an additional matrix for integrative assessment [87,103].

### 7.5. Integrated Diet–Microbiome–Redox–Biomarker Framework

Taken together, Figure 1 and Figure 2 provide a unified conceptual framework linking dietary inputs and microbiome modulation to redox-sensitive signaling pathways and their measurable biomarker outputs. Nutritional factors—including antioxidant micronutrients, polyphenols, selenium sources, and rumen-protected amino acids—modulate Nrf2, NF-κB, and inflammasome activity, thereby influencing systemic and tissue-specific oxidative balance [1,2,3,4,36,40]. Concurrently, diet-induced alterations in the rumen and intestinal microbiome affect endotoxin production, glutathione metabolism, and host inflammatory responses, further shaping redox homeostasis [14,19,22].

**Figure 4:**
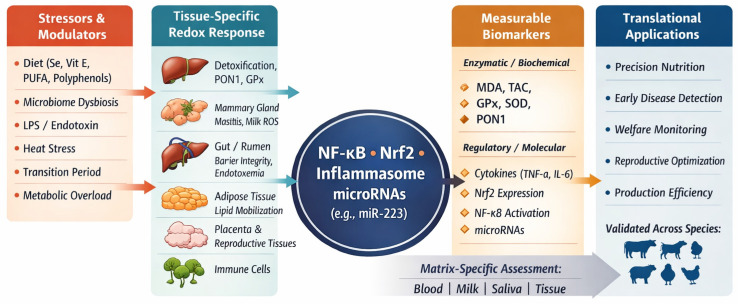
Translational redox biomarker framework across livestock species.

This integrative perspective highlights that oxidative stress biomarkers should not be interpreted in isolation, but rather within the broader context of nutritional status, microbial ecology, tissue specificity, and regulatory signaling networks (Table 2). Such an approach is essential for advancing precision nutrition, precision diagnostics, and targeted antioxidant interventions in modern animal production systems, where oxidative stress serves as both a mechanistic driver and a measurable indicator of health and productivity [5,104]. A comparative overview of major oxidative stress biomarkers, their tissue origin, inflammatory relevance, and species-specific validation is presented in (Table 2, Figure 4).

Dietary factors, microbiome modulation, and environmental stressors induce tissue-specific oxidative responses in key metabolic and immune organs, including the liver, mammary gland, gastrointestinal tract, adipose tissue, and reproductive tissues [30,40]. These responses converge at central redox–inflammatory signaling hubs involving NF-κB, Nrf2, inflammasomes (e.g., NLRP3), and regulatory microRNAs [10,11,12,13,14,15,93]. The resulting biochemical and molecular alterations are reflected in measurable biomarkers (e.g., MDA, TAC, GPx, SOD, PON1, cytokines, gene expression profiles), which can be assessed in various biological matrices such as blood, milk, saliva, and tissues [17,32,111]. Integration of these biomarkers enables precision nutrition, early disease detection, and targeted antioxidant strategies across livestock species [21,26,29].

## 8. Conclusions and Future Directions

Oxidative stress represents a central integrative mechanism linking nutrition, metabolism, immunity, microbiota, and productivity in farm animals. Evidence shows that OS is not merely a passive consequence of ROS overproduction but a regulated, context-dependent process mediated by redox-sensitive signaling pathways, particularly Nrf2/Keap1 and NF-κB (Figure 1). Dietary composition and microbiome-derived metabolites critically modulate these pathways, shaping the magnitude, tissue specificity, and duration of oxidative and inflammatory responses.

The molecular consequences of redox dysregulation are best captured through integrated biomarker panels, rather than single indicators. The Biomarkers–Tissues–Matrices framework (Figure 2, Figure 3 and Figure 4) emphasizes that oxidative stress biomarkers reflect the combined effects of tissue origin, physiological stage, and sampling matrix. Lipid and protein oxidation products, enzymatic and non-enzymatic antioxidants, and inflammation-related mediators provide complementary insights into the animal’s redox status and can inform precision nutrition and management strategies.

Future research should adopt systems-level approaches integrating classical oxidative damage markers with regulatory and signaling-oriented biomarkers, including transcription factors, inflammasome components, and non-coding RNAs, which coordinate redox–inflammatory crosstalk. Longitudinal, multi-omics studies across tissues and developmental stages will enable the identification of early predictive biomarkers of metabolic and immune dysfunction.

From a nutritional perspective, targeted interventions—trace minerals, vitamins, functional amino acids, plant bioactives, and microbiome-directed dietary strategies—should aim to activate endogenous antioxidant defenses rather than merely scavenge ROS, thereby enhancing redox resilience. Expanding non-invasive matrices, such as saliva, milk, urine, and colostrum, will facilitate longitudinal monitoring while improving animal welfare. Standardization of analytical methods and matrix-specific validation are essential to translate oxidative stress research into practical, on-farm diagnostic tools.

## 9. Clinical and Industry Implications

Mechanistic insights into oxidative stress, anchored in diet–microbiome–redox signaling interactions (Figure 1) and supported by integrated biomarker frameworks (Figure 2, Figure 3 and Figure 4), have direct implications for veterinary practice and livestock production:

**- Early Detection of Subclinical Disorders**: Tissue- and matrix-specific biomarkers enable the identification of subclinical metabolic and inflammatory disorders during high-risk physiological stages, such as the periparturient period in cows or gestation/lactation in sows.

**- Precision Diagnostics**: Combining classical oxidative damage markers with enzymatic and inflammation-related indicators enhances risk stratification, enabling targeted interventions for mastitis, metabolic syndrome, reproductive inefficiency, and neonatal health challenges.

**- Non-invasive Monitoring**: The validation of saliva, milk, urine, and colostrum as sampling matrices supports routine, welfare-friendly monitoring in both individual animals and herd-level health programs.

**- Nutrition and Management Strategies**: Optimized diets that modulate redox-sensitive pathways through trace minerals, vitamins, functional amino acids, plant bioactives, and microbiome-targeted interventions can reduce oxidative stress, improve immune function, and enhance production efficiency.

**- Sustainability and Productivity**: Integrating oxidative stress management into routine herd health and nutrition protocols supports sustainable livestock production, improving both product quality and economic performance.

Overall, the combined mechanistic and translational approach outlined in this review provides a framework to bridge molecular redox biology with practical interventions, reinforcing the concept of precision redox management in modern animal production systems.

## Figures and Tables

**Table 1:** Oxidative stress biomarkers in livestock: tissue origin, biological matrices, and translational relevance.

Biomarker	Primary Tissue Source	Biological Matrix	Translational Relevance	References
Malondialdehyde (MDA)	Liver, muscle, mammary gland	Plasma, serum, milk	Widely used indicator of lipid peroxidation; sensitive to metabolic stress during periparturient and early lactation; responsive to antioxidant supplementation	[26,47,60,73]
Thiobarbituric acid-reactive substances (TBARS)	Liver, adipose tissue	Plasma, serum, milk	Reflects cumulative lipid peroxidation; useful for comparative studies but limited specificity	[26,32,111]
F2-Isoprostanes	Systemic (membrane phospholipids)	Plasma, urine, milk	Gold-standard lipid peroxidation marker; high specificity and stability; robust predictor of oxidative stress severity	[51,52,53,54,55,56,57,58]
Protein carbonyls	Liver, immune cells, muscle	Plasma, serum, tissues	Indicator of chronic oxidative stress and irreversible protein damage; linked to inflammation and reduced metabolic efficiency	[17,31]
Xanthine/guanine ratio	Liver, intestinal mucosa	Plasma, urine	Emerging marker of purine oxidation and mitochondrial dysfunction; links redox stress with energy metabolism	[47]
Superoxide dismutase (SOD)	Liver, erythrocytes, immune cells	Plasma, erythrocytes, milk	Reflects enzymatic antioxidant defense; regulated by Nrf2 signaling; responsive to dietary trace minerals	[29,45,49,71]
Glutathione peroxidase (GPx)	Liver, erythrocytes, mammary gland	Plasma, whole blood, milk	Selenium-dependent antioxidant enzyme; marker of functional selenium status and redox resilience	[21,40,69,70]
Catalase (CAT)	Liver, immune cells	Plasma, tissues	Complements SOD and GPx activity; reflects cellular capacity to detoxify hydrogen peroxide	[17,20]
Total antioxidant capacity (T-AOC)/Total antioxidant status (TAS)	Systemic	Plasma, serum, saliva, milk	Integrated measure of redox buffering capacity; useful for monitoring nutritional interventions	[6,41,43,51]
Cytokines (IL-1β, TNF-α, IL-6)	Immune cells, mammary gland	Plasma, milk	Reflect inflammation–redox crosstalk; predictive of disease risk (mastitis, metritis)	[14,19,22]
Acute-phase proteins (haptoglobin, SAA)	Liver	Plasma, serum, milk	Indicators of systemic inflammation and oxidative burden; valuable for herd-level health monitoring	[16,60,98]
microRNA (e.g., miR-223)	Immune cells, mammary epithelium	Plasma, milk exosomes	Regulatory biomarkers integrating redox, inflammation, and epigenetic control; high translational potential	[47]
Salivary antioxidant enzymes	Salivary glands, systemic	Saliva	Non-invasive monitoring of oxidative stress; suitable for longitudinal and welfare-oriented assessments	[35,59,67,69,71]
Colostral oxidative markers	Mammary gland	Colostrum	Reflect maternal redox status and neonatal oxidative exposure; relevant for early-life programming	[87,103]

**Table 2:** Translational relevance of oxidative stress biomarkers across livestock species: tissue origin, inflammatory linkage, and species-specific evidence.

Biomarker	Main Biological Matrix	Predominant Tissue Origin	Redox–Inflammatory Relevance	Species Evidence (Cattle/Pig/Sheep/Poultry)	Key References
Malondialdehyde (MDA)	Plasma, milk, tissues	Systemic lipid peroxidation (liver, mammary gland)	Marker of lipid peroxidation secondary to inflammation and metabolic stress	Cattle ✓/Pig ✓/Sheep ✓/Poultry ✓	[12,13,26,47,50,60,73]
Total Antioxidant Capacity (TAC)	Plasma, serum, saliva	Systemic antioxidant pool	Reflects global redox balance during immune activation	Cattle ✓/Pig ✓/Sheep ✓/Poultry ✓	[6,42,44,52,106]
Glutathione peroxidase (GPx)	Blood, erythrocytes	Liver-dependent selenium metabolism	Detoxifies peroxides; linked to selenium-mediated immune modulation	Cattle ✓/Pig ✓/Sheep ✓/Poultry ✓	[12,21,40,50,69,70]
Superoxide dismutase (SOD)	Blood, tissues	Multiple tissues	Detoxification of superoxide radicals generated during inflammatory ROS burst	Cattle ✓/Pig ✓/Sheep ✓/Poultry ✓	[12,29,45,49,50,71]
Paraoxonase-1 (PON1)	Serum	Hepatic synthesis	Negative acute-phase protein; decreases during systemic inflammation	Cattle ✓/Pig −/Sheep −/Poultry –	[52,60,98,105]
Cytokines (TNF-α, IL-6)	Plasma, milk	Immune cells, mammary gland	Direct mediators of NF-κB-driven inflammation and ROS production	Cattle ✓/Pig ✓/Sheep ✓/Poultry ✓	[12,15,31,43,50,74]
Nrf2 / NF-κB pathway markers	Blood cells, tissues	Tissue-specific (liver, mammary, intestine)	Central regulators of redox–immune signaling crosstalk	Cattle ✓/Pig ✓/Sheep ✓/Poultry ✓	[12,15,44,50,63,72]
microRNAs (e.g., miR-223)	Blood, milk, tissues	Immune and epithelial cells	Regulation of inflammasome and Keap1–Nrf2 axis	Cattle ✓/Pig −/Sheep −/Poultry –	[12,15,50]
Salivary oxidative markers	Saliva	Systemic diffusion	Sensitive to physiological and environmental stress	Cattle −/Pig ✓/Sheep ✓/Poultry –	[6,41,105]
Milk oxidative markers	Milk	Mammary gland	Local oxidative–inflammatory status (mastitis, lactation stress)	Cattle ✓/Pig −/Sheep ✓/Poultry –	[12,50,67,92]

## Data Availability

No new data were created or analyzed in this study.

## Abbreviations

OS	Oxidative stress
ROS	Reactive oxygen species
RNS	Reactive nitrogen species
Nrf2	Nuclear factor erythroid 2–related factor 2
Keap1	Kelch-like ECH-associated protein 1
NF-κB	Nuclear factor kappa-light-chain-enhancer of activated B cells
GPx	Glutathione peroxidase
CAT	Catalase
SOD	Superoxide dismutase
TBARS	Thiobarbituric acid reactive substances
MDA	Malondialdehyde
T-AOC	Total antioxidant capacity
TAS	Total antioxidant status
LPS	Lipopolysaccharide
PUFA	Polyunsaturated fatty acid
MAPK	Mitogen-activated protein kinase
mRNA	Mitochondrial RNA
DNA	Deoxyribonucleic acid
RNA	Ribonucleic acid
